# A computational neural model that incorporates both intrinsic dynamics and sensory feedback in the *Aplysia* feeding network

**DOI:** 10.1007/s00422-024-00991-2

**Published:** 2024-05-20

**Authors:** Yanjun Li, Victoria A. Webster-Wood, Jeffrey P. Gill, Gregory P. Sutton, Hillel J. Chiel, Roger D. Quinn

**Affiliations:** 1 Department of Mechanical and Aerospace Engineering, Case Western Reserve University, 10900 Euclid Avenue, Cleveland, OH 44106 USA; 2 Department of Mechanical Engineering, Carnegie Mellon University, 5000 Forbes Ave., Pittsburgh, PA 15213 USA; 3 Department of Biomedical Engineering, Carnegie Mellon University, 5000 Forbes Ave., Pittsburgh, PA 15213 USA; 4 Department of Biology, Case Western Reserve University, 2080 Adelbert Road, Cleveland, OH 44106 USA; 5 Department of Life Sciences, University of Lincoln, Brayford Pool, Lincoln, Lincolnshire LN6 7TS UK; 6 Department of Neurosciences, Case Western Reserve University, 2080 Adelbert Road, Cleveland, OH 44106 USA; 7 Department of Biomedical Engineering, Case Western Reserve University, 2080 Adelbert Road, Cleveland, OH 44106 USA

**Keywords:** Computational neuroscience, *Aplysia*, Motor control, Sensory feedback, Central pattern generator

## Abstract

Studying the nervous system underlying animal motor control can shed light on how animals can adapt flexibly to a changing environment. We focus on the neural basis of feeding control in *Aplysia californica*. Using the Synthetic Nervous System framework, we developed a model of *Aplysia* feeding neural circuitry that balances neurophysiological plausibility and computational complexity. The circuitry includes neurons, synapses, and feedback pathways identified in existing literature. We organized the neurons into three layers and five subnetworks according to their functional roles. Simulation results demonstrate that the circuitry model can capture the intrinsic dynamics at neuronal and network levels. When combined with a simplified peripheral biomechanical model, it is sufficient to mediate three animal-like feeding behaviors (biting, swallowing, and rejection). The kinematic, dynamic, and neural responses of the model also share similar features with animal data. These results emphasize the functional roles of sensory feedback during feeding.

## Introduction

As an essential motor control task, feeding has been extensively studied in various animals (Avery and You 2012; Vavoulis et al. 2007; Chen 2009). *Aplysia californica*, a species of sea slug, can generate multifunctional feeding behaviors, including biting, swallowing, and rejection (Jing et al. 2004). The neural circuits involved in feeding control contain about 2000 neurons (Moroz 2011), and only a subset of these neurons play the most critical roles (Elliott and Susswein 2002), *Aplysia* uses a relatively small neural network to achieve complex feeding behaviors. Additionally, neurons can be identified across animals (Lu et al. 2013). With large and electrically compact somata, recording or controlling the neurons’ activities through electrodes (Huan et al. 2021) is also possible. These features make *Aplysia* an excellent candidate to research animal feeding. Fully understanding feeding control through studying *Aplysia* can profoundly impact various fields. For instance, it can lead us to discover how animals use small neural circuits to generate behaviors robust to uncertainties and capable of adapting to environmental changes - a critical capability for animals to survive in a dynamic environment (Wolpert and Ghahramani 2000). Furthermore, the knowledge of neuromechanics of *Aplysia* feeding can be applied to solve engineering problems, such as designing and controlling soft robots that can grasp (Mangan et al. 2005; Webster-Wood et al. 2020; Alnajjar and Murase 2008).

A computational model of *Aplysia* feeding can be used as a predictive tool to test hypotheses, thus informing how *Aplysia* achieves multifunctional feeding behaviors. Although existing technologies allow detailed neurophysiological studies of *Aplysia*’s nervous system at a single-cell level, there are still gaps between the observed behaviors and the knowledge of the circuitry. For instance, recent literature identified the existence of feedback pathways and pattern generators in the ganglia of *Aplysia* (Webster-Wood et al. 2020; Elliott and Susswein 2002; Cataldo et al. 2006; Lyttle et al. 2017), but their specific contributions to the overall feeding control remain unclear: Are central pattern generators (CPGs) alone sufficient to generate *Aplysia*-like multifunctional and robust feeding behaviors? Or, is the integration of biomechanics and feedback pathways necessary to more accurately model *Aplysia* feeding control? It is possible to test these hypotheses by using computational models to run numerical simulations and comparing the model outputs with animal data. The comparison results can validate or refine hypotheses and motivate future experimental measurements (Webster-Wood et al. 2020).

Due to the lack of mechanics representing the feeding apparatus, existing computational models of *Aplysia* feeding control have limited predictive ability. Costa et al. (2020) presented a reduced model of an *Aplysia* feeding CPG, which includes CBI-2, a cerebral-buccal interneuron in the cerebral ganglion, and other critical neurons in the buccal ganglion. Cells in the model were represented as Hodgkin-Huxley-type neurons with complex synaptic dynamics. The reduced model was sufficient to generate a variety of motor patterns observed in isolated ganglia of animals. The model did not include cerebral-buccal interneurons (CBIs) that would make it possible to study motor pattern switching. Moreover, since the model focused solely on the nervous system and ignored the peripheral mechanics, it could not demonstrate behavioral responses or reflect sensory feedback’s contribution. The complexity of Hodgkin-Huxley-type models also makes this approach difficult to scale to larger circuits. The computational model presented in Lyttle et al. (2017) considered both peripheral mechanics and neural dynamics of *Aplysia* feeding, allowing the investigation of the effect of sensory feedback. It could flexibly generate biting and swallowing behaviors robust to parameter change. The simulation can be implemented rapidly as the neural dynamics were based on a low-dimensional stable heteroclinic channel (SHC) model. However, the nodes in the model do not have a precise mapping to known neurons. Due to the absence of CBIs, it neither allows egestive behaviors nor active behavioral switching. Webster-Wood et al. (2020) developed a neuromechanical model to study the multifunctional feeding control of *Aplysia*. Following a demand-driven philosophy, the neural dynamics in the model were described as Boolean operations. The incorporated motor neurons and buccal interneurons were driven by proprioceptive feedback, while three cerebral buccal interneurons, CBI-2, CBI-3, and CBI-4, coordinated behavioral switching based on exteroceptive feedback. The model could run several orders faster than real-time, but the neurons performing logic operations were less biologically plausible than the neuronal models in Costa et al. (2020). Thus, the Boolean model cannot fully capture the intrinsic circuit dynamics of animals.

To build an *Aplysia* feeding control model with sufficient predictive ability and low computational complexity, we extended the previous Boolean network model (Webster-Wood et al. 2020) to a Synthetic Nervous System (SNS) version. Like Hodgkin-Huxley-type neurons, the computational capability of SNSs comes from conductance-based mechanisms (Szczecinski et al. 2020), but its computational complexity is less than a spiking model. Following previous literature (Jing and Weiss 2005; Webster-Wood et al. 2020), we organized the SNS neurons into three layers. In the motor neuron and buccal interneuron layers, we divided the neurons into five subnetworks according to their functions. These neurons receive sensory feedback critical for generating adaptive feeding behaviors. In the cerebral ganglion layer, three cerebral-buccal interneurons (CBIs) coordinate behavioral switching based on exteroceptive feedback. The quantitative comparison between the SNS neural models, spiking neural models, and animal data demonstrates that the SNS neurons can capture the features of the neural dynamics observed in animals. We also find the SNS network model can achieve animal-like feeding control when integrated with simplified peripheral biomechanics. These results support the hypothesis that the combination of feedback and a relatively small network in *Aplysia* can generate multifunctional, adaptive, and robust feeding behaviors.

## Methods

We have developed a Synthetic Nervous System (SNS) model for *Aplysia* feeding control, which extends a previous Boolean model of the *Aplysia* neural system (Webster-Wood et al. 2020). Neurons in the model are organized into three layers and five subnetworks according to their functions. In addition, the model integrates sensory feedback loops, which are vital for generating multifunctional and adaptive behaviors.

### Biomechanical model

**Fig. 1:**
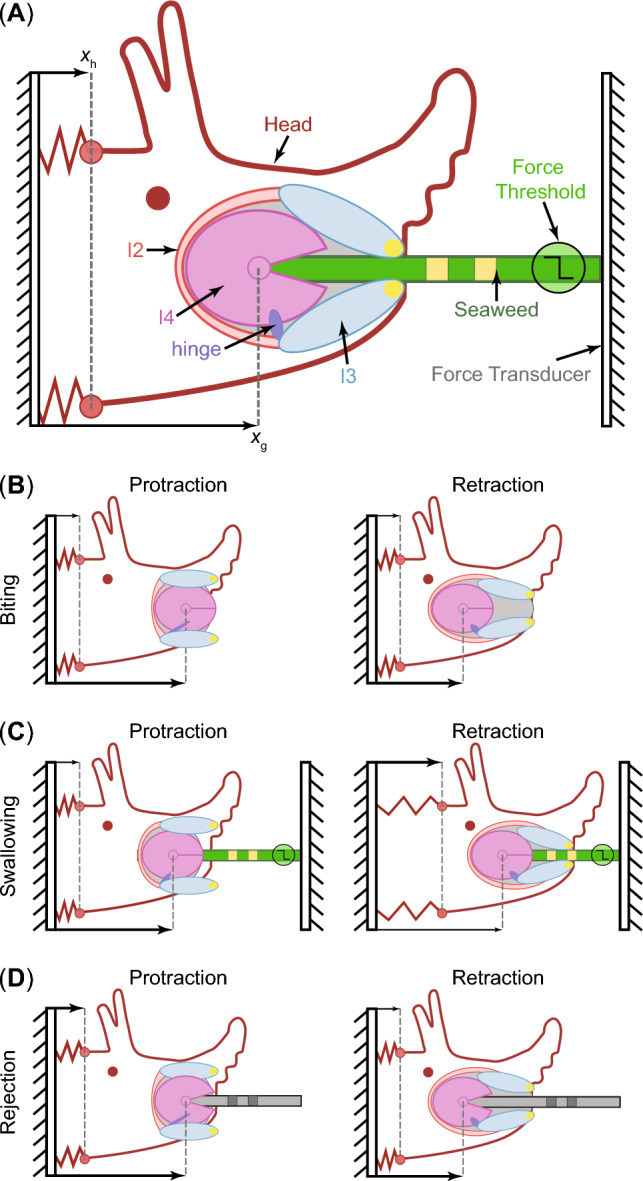
A biomechanical model of *Aplysia* feeding system that can generate multifunctional feeding behaviors. Adapted with permission from Webster-Wood et al. (2020). **A** A schematic showing important elements in the feeding system of *Aplysia*. The grasper in the head can be protracted or retracted by the I2, I3, and hinge muscles. In addition, the retractor muscle I3 contributes to pinching the jaws (yellow circles) onto seaweed, which is critical to prevent seaweed from slipping out during the protraction phase of swallowing. By activating the I4 muscle, the grasper can exert pressure and friction force on the seaweed. We also consider the translation of the head since the head connects to the body by soft tissue. **B** Schematic representation of the model during biting. **C** Schematic representation of the model during swallowing. **D** Schematic representation of the model during rejection. Rejection requires the grasper to be retracted while open and strongly protracted while closed, in contradiction with swallowing. The relative position between the head or grasper and the seaweed or tube is identified by square position markers on the seaweed or tube

This work adopts a simplified biomechanical model of *Aplysia* described in Webster-Wood et al. (2020) (Fig. 1A), which models the translational displacement of the head ($x_{\text {h}}$) and the grasper ($x_{\text {g}}$). The head and grasper are actuated by the I2 protractor muscle, the I3 retractor muscle, and the hinge retractor muscle. The model also includes the I4 muscle and the anterior portion of the I3 jaw muscle, which are responsible for radula closure and jaw closure, respectively. The biomechanical model provides the SNS with proprioceptive feedback of grasper position relative to the head ($x_{\text {g/h}} = x_{\text {g}} - x_{\text {h}}$) and three exteroceptive stimuli, including mechanical and chemical stimulation of the lips, and mechanical stimulation in the grasper. With appropriate control, the demand-driven biomechanical model can generate multiple ingestive and egestive feeding behaviors, including unsuccessful attempts to protract the grasper and grab edible food (biting, Fig. 1B), moving the food inward via the grasper after a successful grasp (swallowing, Fig. 1C), and the expulsion of inedible material out of the mouse using its grasper (rejection, Fig. 1D). Biting is characterized by strong protraction of the grasper, but no food is grasped. In swallowing, food is grasped, and the grasper must be strongly retracted while closed to pull the food into the mouth and weakly protracted while open to re-position the grasper as well as pull more food inwards. The food is a seaweed fixed to the rigid force transducer in the experimental setup (Gill and Chiel 2020). Thus, the retraction when the seaweed is being firmly grasped results in the head being pulled forwards. Rejection requires the grasper to be retracted while open and strongly protracted while closed, in contrast with swallowing. In the experimental setup, the inedible material is generally a tube with no fixation on external objects (Webster-Wood et al. 2020). In Appendix A, we reproduce the calculations for the motion of segments, muscle forces, and contact forces from Webster-Wood et al. (2020) for completeness.

### Synthetic nervous system

**Fig. 2:**
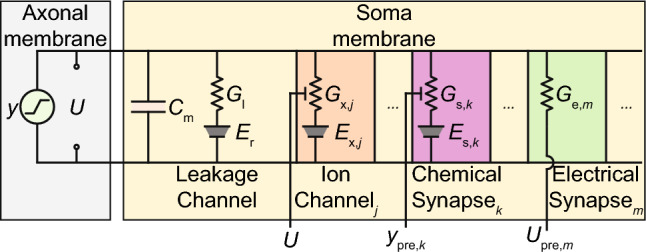
Schematic of neural components in Synthetic Nervous Systems. The model incorporates leak conductance, chemical synapses, electrical synapses, and other ion channels. The currents through these conductances govern the evolution of the membrane potential *U*. Rather than introducing voltage-gated ion channels to explicitly generate spikes, this SNS uses neural activity *y* to reflect the temporal firing frequency. It also neglects the propagation of neural signals along the membrane by treating a neuron as a single-compartment element

Instead of using complex spiking and compartment neuron models, we described the neural dynamics within the framework of the Synthetic Nervous System (Szczecinski et al. 2017b, a). The SNS neuron, as shown in Fig. 2, is a conductance-based, spiking-rate based, and single-compartment neuron model. It balances biological plausibility and computational complexity by considering critical neural components but neglecting the mechanisms underlying the generation and propagation of spikes.

The SNS model in this work uses spiking rate neurons, which means that the relationship between the neural activity $y_i$ and the membrane potential $U_i$ of the *i*th neuron can be expressed as a monotonically increasing activation function $\varphi _i$. The following piecewise linear function is a common selection for $\varphi _i$ (Szczecinski et al. 2017b):

1 $$\begin{aligned} \varphi _{i}(U_{i}) = {\left\{ \begin{array}{ll} 0, &{}\text { if } U_{i} \le U_{\text {lo},i}\\ \frac{U_{i} -U_{\text {lo},i}}{U_{\text {hi},i} - U_{\text {lo},i}}, &{}\text { if } U_{\text {lo},i}< U_{i} < U_{\text {hi},i}\\ 1, &{}\text { otherwise} \end{array}\right. } \end{aligned}$$

where $U_{\text {lo},i}$ and $U_{\text {hi},i}$ are the lower threshold and upper threshold of the activation function, respectively. For B64, B65, B20, and B7 introduced in Sect. 2.3, logistic functions

2 $$\begin{aligned} \varphi _i(U_{i}) = \frac{1}{1 + e^{-(U_{i} - \theta _{i})/\sigma _{i}}} \end{aligned}$$

with parameters $\theta _{i}$ and $\sigma _{i}$ are selected as their activation functions as they empirically facilitate the manual tuning process described in Sect. 2.4. The neural activity $y_i$ can be regarded as an abstraction of the temporal firing rate. If $y_i=0$, it implies that the membrane potential has not achieved the firing threshold, and $y_i=1$ implies the neuron is firing at its maximum frequency.

The equation governing the evolution of $U_i$ in SNSs can be described as (Li et al. 2022):

3 $$\begin{aligned} C_{\text {m},i} \frac{\textrm{d}U_i}{\textrm{d}t} = I_{\text {leak},i} + I_{\text {ion},i} + I_{\text {syn},i} + I_{\text {app},i} \end{aligned}$$

where $C_{\text {m},i}$ is the membrane capacitance. $I_{\text {leak},i}$ and $I_{\text {ion},i}$ define the intrinsic dynamics of the neuron, $I_{\text {syn},i}$ captures the synaptic dynamics, and $I_{\text {app},i}$ is external stimulation. The details of these terms can be found in the following sub-sections.

#### Intrinsic dynamics

In Eq. (3), $I_{\text {leak},i} = G_{\text {l},i}\left( E_{\text {r},i} - U_{i} \right)$, which represents the current flowing through the leakage channel whose conductance is $G_{\text {l},i}$ and leakage reversal potential is $E_{\text {r},i}$. $I_{\text {ion},i}$ represents the currents flowing through other ion channels responsible for strong nonlinear phenomena like plateau potentials and post-inhibitory rebound and is defined as:

4 $$\begin{aligned} I_{\text {ion},i} = \sum _{j} g_{\text {x},ij}A_{ij}^{p_{ij}}B_{ij}\left( E_{\text {x},ij} - U_{i}\right) \end{aligned}$$

where,

5 $$\begin{aligned} \frac{\textrm{d}A_{ij}}{\textrm{d}t}&= \frac{A_{\infty ,ij}(U_{i} ) - A_{ij}}{\tau _{A_{ij}}(U_{i} )} \nonumber \\ \frac{\textrm{d}B_{ij}}{\textrm{d}t}&= \frac{B_{\infty ,ij}(U_{i} ) - B_{ij}}{\tau _{B_{ij}}(U_{i} )} \end{aligned}$$

These ion channels are voltage-gated because each conductance $G_{\text {x},ij} = g_{\text {x},ij}A_{ij}^{p_{ij}}B_{ij}$ is determined by the maximal conductance $g_{\text {x},ij}$ as well as two variables $A_{ij}$ and $B_{ij}$ whose dynamics can be expressed as first order differential equations with membrane-potential-related steady states ($A_{\infty ,ij}$ and $B_{\infty ,ij}$) and time constants ($\tau _{A_{ij}}$ and $\tau _{B_{ij}}$). $E_{\text {x},ij}$ in Eq. (3) is the reversal potential of the corresponding ion channel, and $p_{ij}$ is the activation exponent.

#### Synaptic dynamics

The SNS framework considers synaptic currents flowing through both chemical synapses and electrical synapses, with the total current calculated as,

6 $$\begin{aligned} I_{\text {syn},i}= & {} \sum _{k}{G_{\text {s},ik}\left( E_{\text {s},ik } - U_{i} \right) } \nonumber \\{} & {} + \sum _{m} {G_{\text {e},im} \left( U_{\text {pre},im} - U_{i}\right) } \end{aligned}$$

For the *k*th chemical synapse, $E_{\text {s},ik}$ denotes the reversal potential of the channel. The synaptic conductance $G_{\text {s},ik}$ is determined by a first-order relationship between the activation of presynaptic neuron, $y_{\text {pre},ik}$, and the activation of presynaptic transmitter release, $s_{ik}$, and a first-order relationship between $s_{ik}$ and the activation of the synapse, $r_{ik}$

7 $$\begin{aligned} G_{\text {s},ik}&= g_{\text {s},ik}r_{ik} \nonumber \\ \frac{\textrm{d}r_{ik}}{\textrm{d}t}&= \frac{s_{ik} - r_{ik}}{\tau _{\text {s},ik2}} \nonumber \\ \frac{\textrm{d}s_{ik}}{\textrm{d}t}&= \frac{\alpha _{ik}\left( y_{\text {pre},ik}\right) - s_{ik}}{\tau _{\text {s},ik1}} \end{aligned}$$

where $g_{\text {s},ik}$ is the maximal conductance of the channel. $\tau _{\text {s},ik1}$ and $\tau _{\text {s},ik2}$ are activation constants determining whether the synapse transmission is fast or slow. In this work, $\alpha _{ik} \left( y_{\text {pre},ik}\right) = y_{\text {pre},ik}$ except for those synapses with the interneuron B4 (see Sect. 2.3.2) as their presynaptic neurons. To account for variable bursting intensities of B4 observed in our animal experiments, we set $\alpha _{i\text {B4}} \left( y_{\text {B}4}\right) = \frac{1}{1 - \beta _{i\text {B}4}} \max (0, y_{\text {B}4} - \beta _{i\text {B}4})$ so that B4 activity $y_{\text {B}4}$ will not influence the postsynaptic neuron until it is greater than the corresponding synaptic threshold $\beta _{i\text {B}4}$. For the *m*th electrical synapse, $G_{\text {e},im}$ is the constant synaptic strength, and $U_{\text {pre},im}$ is the membrane potential of the corresponding presynaptic neuron.

#### External stimulation

$I_{\text {app},i}$ in Eq. (3) defines an optional external stimulus current, which can represent injected current via an electrode or current from feedback loops. In this work, sensory feedback signals were implemented in the form,

8 $$\begin{aligned} I_{\text {app},i} = \sum _{n} \xi _{in}\max \left( \epsilon _{in} (x_{in} -S_{in}),0\right) \end{aligned}$$

where *n* is the index of the feedback loop, $x_{in}$ is the feedback input which represents $x_{\text {g/h}}$ (see Sect. 2.1) for a proprioceptive feedback loop or any external stimulus introduced in Sect. 2.3.3, $\xi _{in}$ and $S_{in}$ are the feedback gain and the threshold of the feedback input, respectively (excitatory feedback if $\xi _{in} > 0$, inhibitory feedback if $\xi _{in} < 0$). $\epsilon _{in} \in \{-1,1\}$ determines whether the feedback loop is triggered on a rising or falling edge.

### Neural circuitry model

**Fig. 3:**
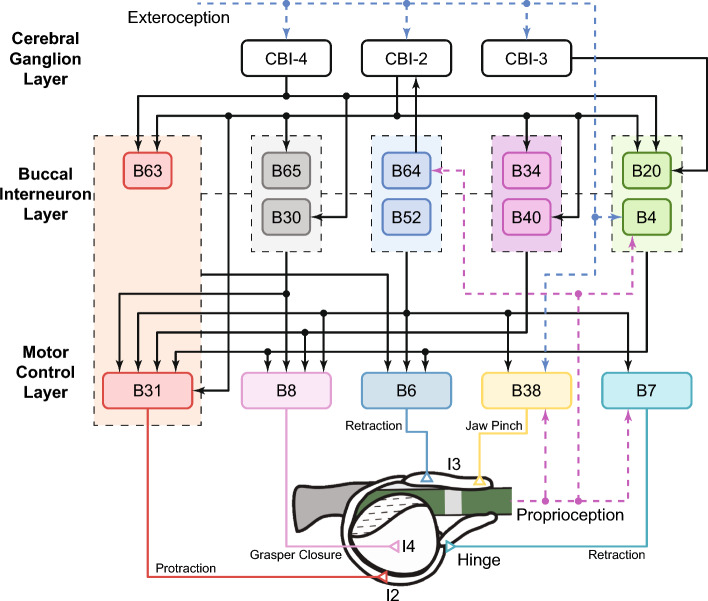
The neural circuitry model adapted with permission from Li et al. (2022). Neurons in the model are organized into three layers. In the motor control and buccal interneuron layers, neurons are further divided into five subnetworks according to their functions. Neurons in the same network are indicated by the same color. The cerebral ganglion layer contains cerebral interneurons for behavioral switching and coordination. Cross-layer synaptic connections are shown as bold black arrows. Dashed black lines represent inter-layer connections. Sensory signals, including proprioceptive and exteroceptive feedback, may be provided by additional sensory neurons or interneurons

Using the SNS framework, we developed a neural circuitry model of *Aplysia* feeding control. The model incorporates known synaptic connections and plausible feedback signals from the peripheral biomechanics (Fig. 3). Previous work (Jing and Weiss 2005; Webster-Wood et al. 2020) suggests that the neural network for *Aplysia* feeding control may have a hierarchical architecture. Following this perspective, we organized neurons in the model into three layers and five subnetworks based on their functional roles in producing multifunctional feeding behaviors. The motor control layer contains motor neurons innervating the muscles. The subnetworks of the buccal interneuron layer receive sensory feedback and descending signals to coordinate the timing of motor control patterns. The cerebral ganglion layer receives exteroceptive signals and controls the type of feeding behavior generated by modulating neurons in the buccal interneuron layer.

#### Motor control layer

The motor control layer models five motor neurons innervating key musculature in the *Aplysia* feeding apparatus: B31 innervates the I2 protractor muscle for protracting the grasper (Hurwitz et al. 1996); B6 innervates the I3 retractor muscle for retracting the grasper (Morton and Chiel 1993a); B8 innervates the I4 muscle, used in this model for closing the grasper (Morton and Chiel 1993a); B38 innervates the anterior portion of the I3 muscle for pinching the jaws (McManus et al. 2014); B7 innervates the hinge muscle for facilitating initial retraction (Sutton et al. 2004). These neurons receive excitatory and inhibitory synaptic inputs from the buccal interneuron layer; some (B7 and B38, (Webster-Wood et al. 2020)) are also mediated by sensory feedback (Fig. 3). Detailed synapses and feedback pathways to motor neurons can be found in Tables 3 and 5.

For simplicity, we only considered the most critical motor neurons and interneurons for multifunctional feeding control. Therefore, neurons in this model can be regarded as abstractions of larger neuron pools in the animal. For example, B31 here represents the motor neuron pool B31/B32/B61/B62 innervating the I2 muscle (Hurwitz et al. 1994), and B6 represents the motor neuron pool B6/B9/B3 innervating the I3 muscle (McManus et al. 2014). Furthermore, we assumed the feedback stimuli directly acted on the neurons, although sensory neurons and interneurons exist in the actual feedback loops. Additional elements in these neuron pools and feedback loops could be easily added to the model as deemed necessary to match the kinematic and dynamic behavior in the animal.

#### Buccal interneuron layer

**Fig. 4:**
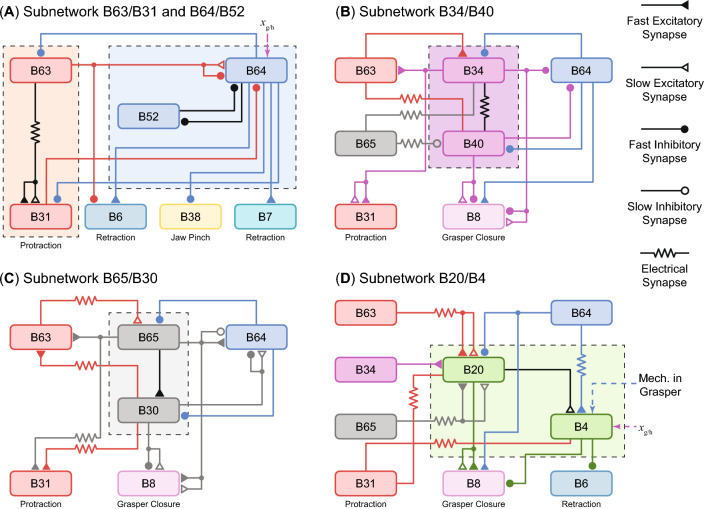
Schematic of the buccal interneuron layer adapted with permission from Li et al. (2022). This layer contains five subnetworks critical for the multifunctional feeding control of *Aplysia*. Different combinations of these subnetworks can generate behavioral patterns with different features. (**A**) Pathways in the subnetworks B63/B31 and B64/B52. (**B**) Pathways in the subnetwork B34/B40. (**C**) Pathways in the subnetwork B65/B30. (**D**) Pathways in the subnetwork B20/B4. Neurons in different subnetworks are highlighted in different colors, and each subnetwork is covered by a dashed and color-coded rectangle. The inter-subnetwork synaptic connections are color-coded according to their presynaptic neurons, while the intra-subnetwork connections are color-coded by black

We modeled nine buccal interneurons in the buccal interneuron layer and organized them into five subnetworks (B63/B31, B64/B52, B34/B40, B65/B30, B20/B4) based on their functional roles in biting, swallowing, or rejection (Fig. 4). The control subnetworks are taken from Jing and Weiss (2005, 2001) and Costa et al. (2020) with modifications and the inclusion of sensory feedback (Webster-Wood et al. 2020). Each subnetwork receives behavioral commands from the cerebral ganglion layer and modulation from other subnetworks; some neurons (in particular, B64 and B4) also receive sensory feedback to regulate the timing of their activation. When a subnetwork is active, it excites or inhibits a specific set of motor neurons to control the innervated muscles. In our model, several subnetworks can be active simultaneously, indicating that the stimulation of a motor neuron is a net result of all active subnetworks innervating it.

**The B63/B31 and B64/B52 subnetworks** (Fig. 4A) are the main subnetworks realizing grasper protraction and retraction, respectively. Whether in ingestive or egestive behaviors, the first essential function of *Aplysia* feeding control is robustly generating back-and-forth movements of the grasper. The strong excitatory synaptic connections from B63 to B31 guarantee that motor neuron B31 can be active with B63 (Hurwitz et al. 1997). Due to their highly correlated firing patterns, B63 and B31 have been previously viewed as a single functional unit (Hurwitz et al. 1997). Similarly, they are grouped into a single subnetwork in this work despite being from different layers. Subnetwork B64/B52 is responsible for realizing the shift from protraction to retraction and back. B64 is a buccal interneuron that fires throughout the retraction phase (Hurwitz and Susswein 1996). Its activation is initiated by a slow excitatory synapse from B63 (Costa et al. 2020; Zhang et al. 2020) and maintained even beyond the termination of the protraction phase due to its intrinsic dynamics (see Sect. 3.1). The existence of proprioceptive input further enables its activation to be adaptive to the external load (Webster-Wood et al. 2020; Borovikov et al. 2000). B64 can mediate I3 muscle contraction and grasper retraction through an excitatory connection to motor neuron B6 (Elliott and Susswein 2002). To guarantee the protraction-retraction shift, it has abundant inhibitory connections to those neurons active during the protraction phase (i.e., B30, B34, B40, B65, B63, B31, B20) (Hurwitz and Susswein 1996). On the other hand, B52 is an interneuron that can produce post-inhibitory rebounds (PIRs) through a low threshold sodium channel (see sect. 3.1). Mutual inhibitory synaptic connections between B64 and B52 allow inhibition from B64 to elicit PIRs in B52 (Costa et al. 2020), which in term guarantees the termination of the retraction phase and extends the protraction phase (Cataldo et al. 2006; Nargeot et al. 2002).

**The B34/B40** (Fig. 4B) **and B65/B30 Subnetworks** (Fig. 4C) are responsible for mediating radula closure during the retraction phase and regulating the length of protraction. They are thus critical for generating ingestive feeding behaviors. Subnetwork B34/B40 plays a key role in producing biting (Jing et al. 2003). Receiving excitatory synapses from B63/B31 and inhibitory synapses from B64 (Hurwitz et al. 1997; Jing and Weiss 2002), it is active in the protraction phase and serves a dual purpose. On the one hand, the slow excitatory synapses from B34 and B40 to the motor neuron B8 have functional roles in increasing the excitability of the postsynaptic neuron (Hurwitz et al. 1997; Jing and Weiss 2002). This increase in excitability allows B8 to generate stronger activation for radula closure during the retraction phase. On the other hand, the B34/B40 subnetwork delays the onset of the retraction phase and thus prolongs the protraction phase through monosynaptic inhibitory connections to B64 (Jing et al. 2003). Radula closure during the retraction phase and a relatively longer protraction phase are two features of biting that enable an animal to grasp food outside the mouth (Jing et al. 2004). Subnetwork B65/B30 plays a key role in producing swallowing (Jing et al. 2004). B65 (Kabotyanski et al. 1998) and B30 (Jing et al. 2004) are protraction neurons that receive excitatory inputs from B63/B31 and inhibitory input from B64. They also innervate B8 through slow excitatory synapses to mediate the radula closure during the retraction phase (Jing et al. 2004). In contrast to B34/B40, B65/B30 has excitatory connections to B64 that accelerate the onset of the retraction phase and shorten the protraction phase (Kabotyanski et al. 1998; Jing et al. 2004). The length of the protraction phase distinguishes swallowing from biting.

**The B20/B4 subnetwork** (Fig. 4D) controls the phase in which radula closure occurs, thus mediating the switch between ingestive and egestive behaviors (Jing and Weiss 2001). B20 is a protraction interneuron because the excitation from other protraction interneurons, such as B34, B31, and B65, and inhibition from the retraction interneuron B64 constitute the main part of its input (Jing and Weiss 2001). Monosynaptically exciting B8, it provides strong stimulation to the radula closure motor neuron during the protraction phase. In addition, it makes slow monosynaptic chemical connections to the retraction interneuron B4. The excitatory synapses, whose effects exist even after B20 stimulation, functionally increase the excitability of B4, allowing the excitatory synapse from B64 to elicit strong activation in B4 during the retraction phase (Jing and Weiss 2001). B4 prevents the radula closure motor neuron from firing during retraction through an inhibitory synapse to B8 (Jing and Weiss 2001; Kabotyanski et al. 1998). By overwriting the inhibitory and excitatory stimulation from other subnetworks, respectively, B20 and B4 enable the radula closure during the protraction phase, a critical feature of egestive behavior such as rejection. Furthermore, sensory feedback is incorporated to excite B4 so that its maximal activation occurs at the end of the protraction phase and the onset of the retraction phase, as observed in animals (Morton and Chiel 1993b). With the inhibitory synapse to B6 (Gardner 1971), the B4 activation can delay the onset of the activity in the I3 muscle, ensuring the contraction of the jaw muscle does not force the grasper to close or pull the inedible food inward during rejection (Ye et al. 2006).

#### Cerebral Ganglion layer

**Fig. 5:**
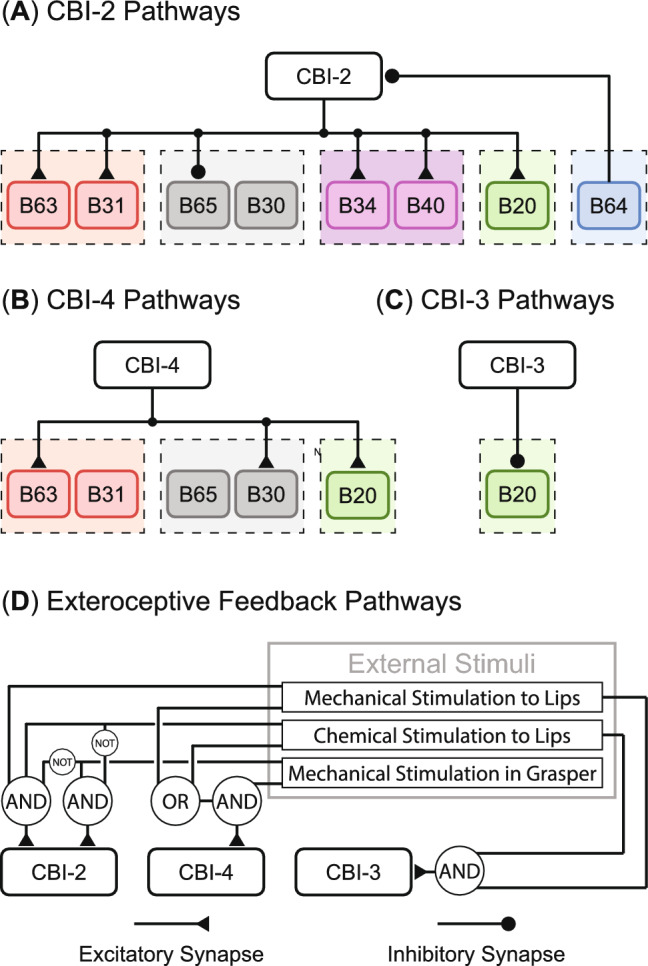
Schematic of the cerebral ganglion layer. Cerebral-buccal interneurons in this layer are command-like neurons that excite or inhibit different subnetworks in the lower buccal ganglion layer. They are driven by external chemical and mechanical stimuli. **A** CBI-2 pathways. **B** CBI-4 pathways. **C** CBI-3 pathways. **D** Exteroceptive feedback pathways (Adapted with permission from Webster-Wood et al. (2020))

The cerebral ganglion layer mediates the switch between different behaviors by selectly stimulating the subnetworks introduced in Sect. 2.3.2 (Fig. 5). In this layer, we modeled three cerebral buccal interneurons confirmed as critical components for behavioral selection. CBI-2 (Fig. 5A) monosynaptically excites B63, B31 (Hurwitz et al. 2003), B34 (Sńchez and Kirk 2000), and B40 (Jing and Weiss 2002), but inhibits B65 (Jing and Weiss 2005). Thus, it strongly recruits subnetwork B63/B31 and B34/B40. In contrast, another command-like neuron in this layer, CBI-4 (Fig. 5B), recruits subnetworks B63/B31 and B65/B30 by exciting B63 and B30, respectively (Jing et al. 2004). By inhibiting B20, CBI-3 (Fig. 5C) permits shifting the phase in which the radula closes with respect to protraction-retraction (Jing and Weiss 2001). The combinational stimulation of the CBI neurons allows the production of different behaviors with distinguishable features. For example, the behavior elicited by CBI-2 and CBI-3 tends to be ingestive and has a longer protraction phase (biting). On the other hand, the behavior elicited by CBI-4 and CBI-3 is still ingestive but has a longer retraction phase (swallowing). If CBI-3 is silent during the actions of CBI-2 or CBI-4, the elicited behavior becomes egestive (rejection).

The cerebral ganglion layer coordinates behavioral switching based on exteroceptive feedback. Referring to existing literature (Gill and Chiel 2020; Webster-Wood et al. 2020), animals generate biting if they are presented with edible food such as strips of dried nori (mechanical and chemical stimulation simultaneously act on lips while no mechanical stimulation acts on graspers). The behavior immediately switches to swallowing once animals successfully grasp food (all three stimuli exist). If they detect inedible objects (either chemical or mechanical stimulation to lips is missing, and the mechanical stimulation in the grasper is maintained), rejection will be initiated to push objects out. Following the same logic formulations presented in Webster-Wood et al. (2020), we applied excitatory or inhibitory stimuli to the CBI neurons so that the neuromechanical model could handle behavioral transitions like animals (Fig. 5D). In a specific behavior, CBI-3 and CBI-4 remain tonic or silent as their activations are totally determined by external stimuli. CBI-2, in contrast, also receives inhibition from the interneuron B64 (Fig. 5A). Since B64 is activated during the retraction phase, the inhibition results in rhythmic output of CBI-2 during biting and rejection.

### Implementation and parameter tuning

We implemented the neuromuscular model in the Matlab Simulink environment (R2023a) with the variable-time-step ode45 solver (max. step size of 1 ms, relative and absolute error tolerance of $1\times 10^{-3}$). The circuitry model has 486 parameters (120 for intrinsic dynamics, 298 for chemical synapses, 28 for electrical synapses, and 24 for proprioceptive feedback pathways, 16 for exteroceptive feedback pathways, Appendix B). The simulation runs 1.2 times faster than real-time on a desktop computer (128 GB RAM, 3.00 GHz CPU).

We developed the details of SNS neural dynamics based on recent computational models (Costa et al. 2020; Cataldo et al. 2006; Vavoulis et al. 2007). Using the SNNAP platform, Costa et al. (2020) built a reduced model of the CPG in the isolated ganglia of *Aplysia* for the generation of feeding-related buccal motor patterns. The neurons in the reduced model incorporate slow conductances that mediate intrinsic dynamics and fast conductances that mediate spiking. We modeled these slow conductances in the SNS neurons and fine-tuned the parameters in the B64, B4, and B52 models, allowing them to capture the firing thresholds and current-activation curves observed in animals. Specifically, for B64, we ran the CPG network model in (Costa et al. 2020), recorded the time series of the net synaptic current going into B64, $\textbf{I}_{\text {B}64}$, and the normalized firing rate of B64, $\hat{\textbf{y}}_{\text {B}64}$. We then optimized the parameters in the SNS model of B64 to minimize the following cost function, *J*:

9 $$\begin{aligned} J = \text {xcorr}\left( \hat{\textbf{y}}_{\text {B}64},\textbf{y}_{\text {B}64}\right) \end{aligned}$$

where xcorr denotes the cross-correlation measure, and $\textbf{y}_{\text {B}64}$ is the activity generated by the SNS model of B64 with the previously recorded input current $\textbf{I}_{\text {B}64}$. For B4 and B52, we optimized their neural parameters to minimize the root-mean-square error (RMSE) between the current-activation curves produced by the SNS neurons and those observed in animals. For those neurons that do not exist in Costa et al. (2020), we assumed they had similar capacitance, leak conductance, and resting potential values as the other neurons.

In addition, we hand-tuned the parameters in synaptic connections and feedback pathways so that the integrated neuromuscular model could qualitatively generate *Aplysia* feeding behaviors. The computational model presented in (Costa et al. 2020) also included synaptic mechanisms such as synaptic weights, time constants, and complex synaptic plasticity. However, these mechanisms and parameters in chemical synapses are based on spiking. Therefore, they cannot be directly used in a network model with spiking-rate based neurons. Costa et al. (2020) also neglected the proprioceptive and exteroceptive feedback in their model to focus on the motor programs in isolated ganglia. In this work, we tuned the synaptic and feedback parameters in the SNS network by trial and error until simulations reproduced animal-like feeding behaviors, and the timing of ingestive behaviors shared similar features with animal data.

### Availability of data and model code

The Simulink model and source code are available at https://github.com/CMU-BORG/C3NS-IRG3-Simulink-Modular-Model. Archived code is available through Zenodo (doi: 10.5281/zenodo.10228820)

## Results

After implementing the model, we performed several tests to compare model output with animal data to validate the model. We first show that SNS neurons are capable of reproducing the intrinsic dynamics of specific *Aplysia* neurons with reported dynamics in the literature: B64, B4 (Hurwitz and Susswein 1996), B34 (Hurwitz et al. 1997), and B52 (Plummer and Kirk 1990). We then confirm that the proposed neural control circuitry model can produce multifunctional feeding behaviors of *Aplysia californica* when connected with the biomechanical model. To further assess the model, we compare the kinematics and kinetics responses of the neuromechanical model with animal data. In response to external sensory cues, the model can also switch between ingestive and egestive behaviors.

### Intrinsic neural dynamics

**Fig. 6:**
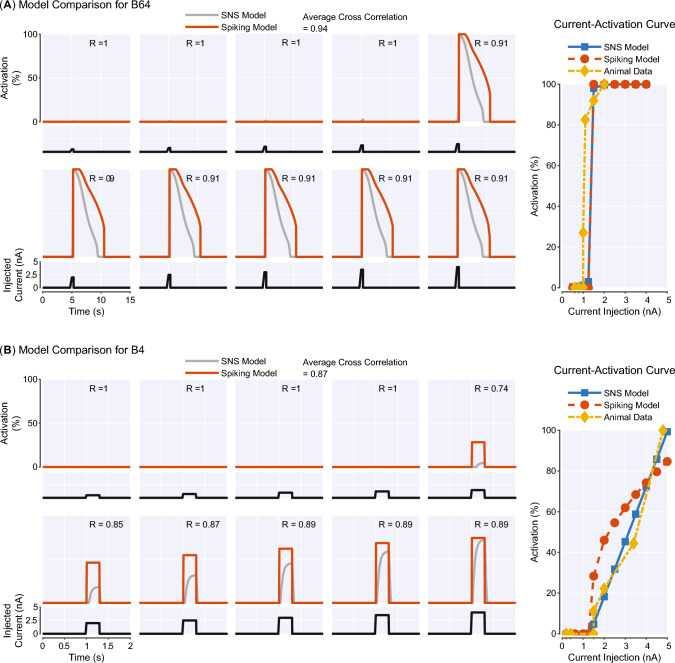
Comparison between the Synthetic Nervous System model, a spiking model, and animal data for neurons **A** B64 and **B** B4. For each neuron, the left graph shows 10 brief input responses produced by the SNS model and a spiking model in Costa et al. (2020). The activation of the spiking model is its instantaneous firing rate normalized to the peak value we observed in the simulation (12 Hz for B64, 15 Hz for B4). As a measure of similarity, cross correlation (*R*) between the SNS model response and spiking model response is shown for each brief input test. The averaged cross correlation is also given at the top. The right graph shows the current-activation relationship of the corresponding neuron obtained from the SNS model, spiking model and animal subjects

**Fig. 7:**
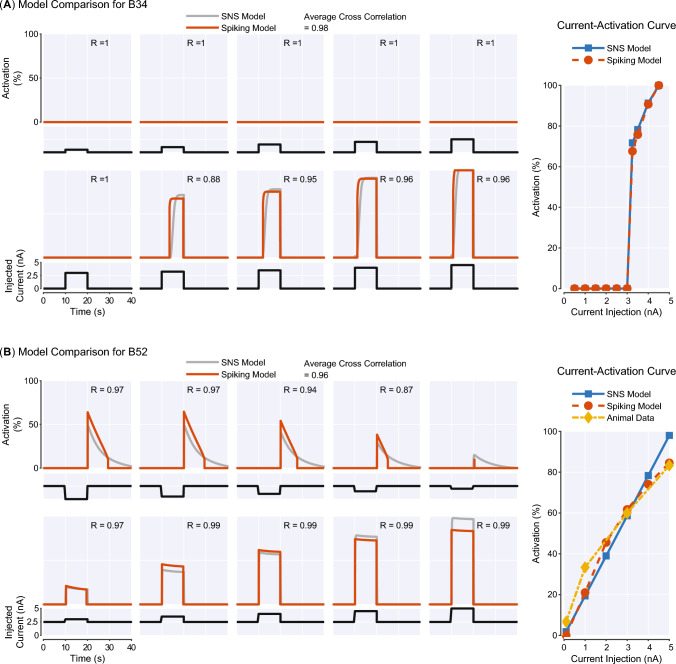
Comparison between the Synthetic Nervous System model, a spiking model, and animal data for neurons **A** B34 and **B** B52. Similar with Fig. 6, both sustained input test and current-activation curve are shown for each neuron. The activation of the spiking model is its instantaneous firing rate normalized to the peak value observed in the simulation (10 Hz for B34, 15 Hz for B52). Cross correlation (*R*) is used to compare the response similarity for input tests

The SNS circuitry can generate animal-like neural dynamics, although the output of neurons is activation instead of spikes. To assess the intrinsic dynamics of our SNS model, we compared the output of selected SNS neurons to a prior spiking model introduced in Costa et al. (2020) and to animal data (Figs. 6, 7). The output of a spiking neuron model is a temporal sequence of action potentials. To determine its activation, we calculated interspike intervals (ISIs), the intervals between action potentials. We could then define its instantaneous firing rate as the reciprocal of ISIs and its activation as the normalized instantaneous firing rate. Four neurons, B64, B4, B34, and B52, were chosen for comparison due to their unique intrinsic dynamics, functional importance, and availability of current-activation data in the prior literature.

The retraction interneuron B64 has been identified as a critical component for terminating the protraction phase and maintaining the retraction phase (Hurwitz and Susswein 1996) in *Aplysia* feeding control. During the retraction phase, it expresses a sustained regenerative firing and inhibits cells that are active during protraction. The SNS and spiking models of B64 incorporate a fast $\text {Na}^+$ channel and a slow $\text {K}^+$ channel to reflect its intrinsic dynamics. We find that brief depolarization (0.3 s) can elicit sustained bursts in both neuronal models (Fig. 6A). Furthermore, once the current level in both models is above a threshold (1.25 nA), the firing frequencies of bursts are not strongly affected by the injected current. These features, including spontaneous bursts and highly nonlinear current-activation relationship, are matched to reported animal data (Hurwitz and Susswein 1996). In contrast, B4 is an interneuron responsible for generating rejection behaviors with a relatively linear current-activation relationship (Hurwitz and Susswein 1996). The SNS model of B4 reflects these dynamics because no additional conductances exist in its membrane. The thresholds of firing (Fig. 6B) in the SNS model (1.20 nA) and spiking model (1.25 nA) lie within the range found in animal experiments (1.0–1.5 nA) (Hurwitz and Susswein 1996).

**Fig. 8:**
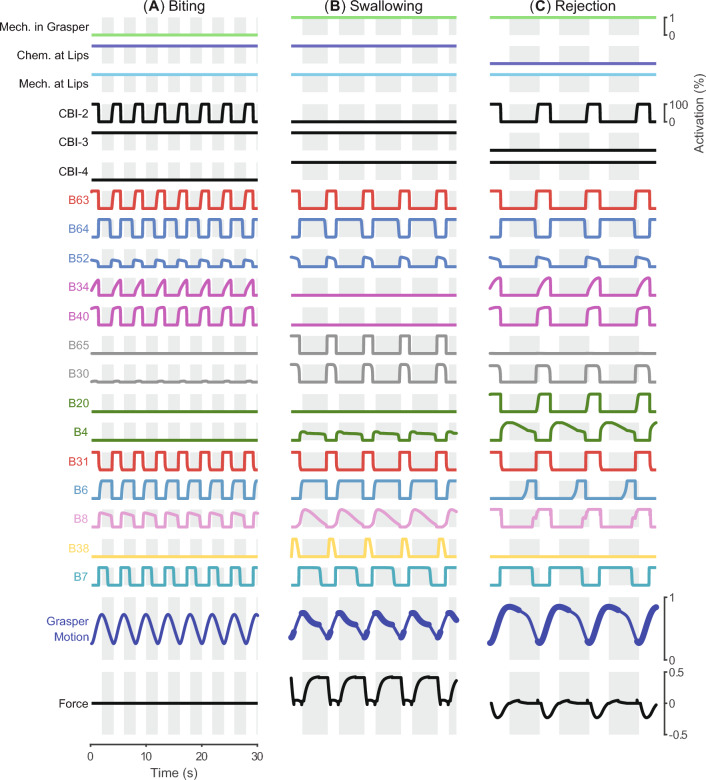
The integration of the SNS model and a simplified periphery model can produce animal-like kinematics, kinetics, and neural activities for three *Aplysia* feeding behaviors: **A** biting, **B** swallowing, and **C** rejection. In biting, the motion of the periphery is relatively fast. No force is applied to the seaweed as the coefficients of friction are manually set to zero because biting is a failure to grasp food. In swallowing, a protraction phase is followed by a relatively longer retraction phase, making the total duration of the feeding increase. The coefficients of friction are restored so that the simultaneous activation of B8 and B6 results in a strong positive force on the seaweed during the retraction phase. In rejection, the total duration of the behavior becomes even longer. The coincidence between the protraction phase and grasper closure is mediated by the B20/B4 module. Positive forces here indicate the seaweed is being pulled inward, while negative forces indicate the neuromechanical model is pushing the food out. Shaded backgrounds indicate retraction phases. Thickening of the grasper motion trace represents the position of the grasper when closing pressure would be large enough to avoid slip

B34 is a protraction interneuron vigorously active during CBI-2-elicited behaviors (Hurwitz et al. 1997). During depolarization, the neuron can start firing only after a slow $\text {K}^+$ current inactivates (Hurwitz et al. 1997). Therefore, the spiking model and SNS model of B34 incorporate a slow $\text {K}^+$ channel to account for this mechanism. When injected with sustained input currents, both models produce strong activation after 2 s (SNS model: 3.3 s, spiking model: 2.0 s), and the activation can be seen only after the level of input current achieves high values (3.25 nA) (Fig. 7A). These results are consistent with the observation that B34 is depolarized in phase with B31/B32 in many buccal motor programs, but does not fire. Additionally, the depolarization of B34 elicited firing after a long delay is also observed in the animal data (Hurwitz et al. 1997). Another interneuron with distinctive physiological properties that plays a functional role in feeding control is B52. This neuron can display a burst of action potentials on rebound from hyperpolarization, which is critical for guaranteeing the termination of the retraction phase (Plummer and Kirk 1990). Post-inhibitory rebound can be produced by several mechanisms, including hyperpolarization-activated cation current (Jones and Thompson 2015). Inspired by the model in Costa et al. (2020), we added a hyperpolarization-activated $\text {Na}^+$ conductance to the SNS model of B52. This channel has slow kinetics and can produce a slow-decaying inward cation current to polarize the neuron after a hyperpolarization. We find that both SNS and spiking models of B52 can generate post-inhibitory rebound with the slow $\text {Na}^+$ conductance (Fig. 7B).

### Multifunctional and robust feeding control

By combining the SNS neural circuitry with the simplified peripheral biomechanics (see Sect. 2.1), we obtained a neuromuscular model of *Aplysia* feeding sufficient to qualitatively generate two ingestive behaviors (biting and swallowing, Fig. 8A, B) and one egestive behavior (rejection, Fig. 8C). To elicit biting in simulation, we applied both chemical and mechanical stimulation to the lips. We also removed the interactive mechanism between the periphery and the seaweed. Since biting is defined as failed attempts to grasp food, the seaweed should experience no friction force in this behavior. The model generates a rhythmic behavior with biting-like features such as similar protraction and retraction durations (Fig. 8A). Interneurons B34 and B40 are strongly activated, while B65, B30, B20, and B4 show low activations due to the activation pattern of the CBI neurons. If we further apply mechanical stimulation to the grasper and restore the interaction between the periphery and the environment, the model generates swallowing-like behaviors (Fig. 8B). As observed in animal swallowing, the protraction duration produced by the model is shorter than the retraction duration. B65/B30, rather than B34/B40, are activated during the protraction phase. Mediated by B8, the grasper closer muscle I4 closes in-phase with the retraction phase, exerting a large positive (ingestive) force on the seaweed to pull the food into the buccal mass. In contrast, if we remove the chemical stimulation applied to the lips to indicate the presence of an inedible material, the model starts a rejection-like behavior with a longer cycle duration than the aforementioned ingestive behaviors (Fig. 8C). The behavior is egestive because the alternating activations of B20 and B4 result in a negative (egestive) force on the seaweed during the protraction phase.

**Fig. 9:**
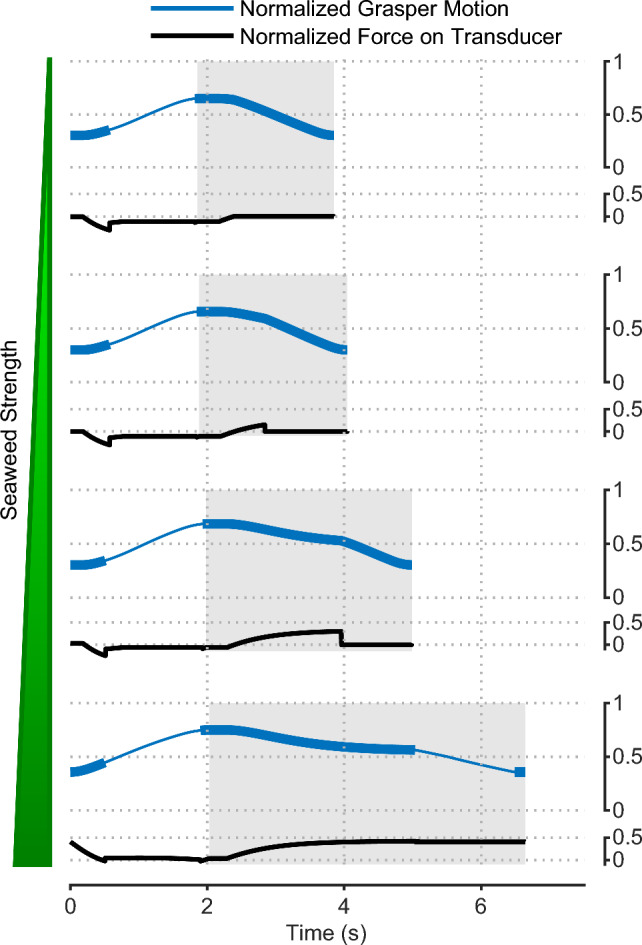
Normalized grasper motion and measured force on the force transducer during a single swallowing cycle with varying seaweed strength thresholds. Thickening of the grasper motion trace represents the position of the grasper when closing pressure would be great enough to avoid slip. Shaded backgrounds indicate retraction phases. Seaweed strength thresholds indicated by vertical green line widths increase from 0.0, where seaweed breaks early in the retraction phase (force on the transducer quickly drops to zero), to 0.45, at which point the seaweed does not break. The cycle period of swallows increases with increasing seaweed strength

Similar to animals, the neuromuscular model exhibits robustness within feeding control. Robustness enables animals to maintain fitness with respect to perturbation and thus is critical for survival in an ever-changing environment. It can be observed when animals attempt to swallow seaweeds with varying mechanical strength that animals tend to increase the duration of swallows, particularly the retraction phase, so that they can generate a strong enough retraction to feed on food with increasing mechanical load. In the simulation, we can modify the mechanical load by adjusting the seaweed strength parameter, controlling the force threshold at which the seaweed breaks. As observed in behaving animals, increasing the value of this parameter leads to a longer swallowing duration and retraction phase (Fig. 9). Initial negative forces during protraction are consistent with those observed in the animal (Webster-Wood et al. 2020). As the model parameters were not explicitly optimized to reproduce this phenomenon, the robustness demonstrated here is an emergent property of the feedback mechanisms. When feedback pathways in the model are removed, the cycle duration remains constant at 11.60 s for different levels of mechanical load. This duration is longer than that observed when sensory feedback is present and the seaweed is unbreakable.

### Ingestive response comparison

**Fig. 10:**
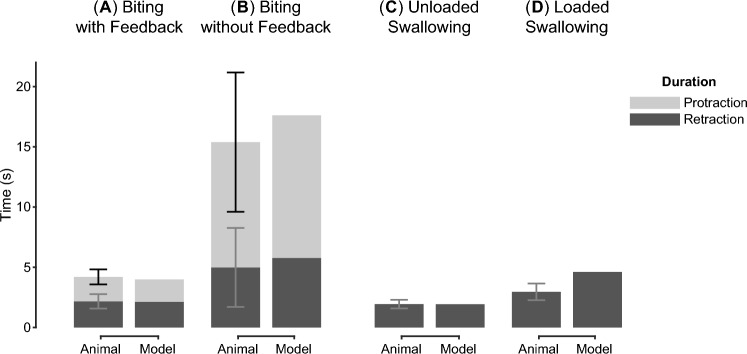
Comparison between behavioral durations of the animal data and the neuromuscular model. The model validation was conducted based on four experiments: **A** in vivo biting (unpublished data from Cullins et al. (2015)), **B** ingestive patterns generated by isolated ganglion (unpublished data from Cullins et al. (2015)), **C** swallowing loose seaweed (Gill and Chiel 2020) and **D** swallowing unbreakable seaweed (Gill and Chiel 2020). **A** In in vivo biting, the intact feedback pathways promoted short total durations with similar protraction and retraction durations. **B** In isolated ganglion preparations, all feedback pathways were removed. The ingestive patterns generated by animals have total durations 3.68 times longer than the in vivo biting. The majority of the increase occurs in the protraction phase (5.12 times longer than **A**) rather than the retraction phase (2.30 times longer than **A**). The biting responses of the model with and without feedback pathways share similar characteristics with the animal data. **C** In swallowing loose seaweed, the freely-moving food exerted little load on the radula. **D** In swallowing unbreakable seaweed, the load prevented animals from pulling the food inward, leading to a longer behavior duration than the unloaded case. The increased load also induced longer swallowing in the simulation. Error bars indicate SD

The neuromuscular model shares similar behavioral durations with animals for *in vivo* biting and *in vitro* ingestive patterns in isolated ganglia (Fig. 10). Intact animals generate biting with relatively short cycle durations (4.26 ± 0.95 s, Fig. 10A), as well as similar protraction duration (2.03 ± 0.62 s) and retraction duration (2.17 ± 0.60 s). The biting behavior produced by the neuromuscular model has similar protraction duration (1.84 s, Fig. 10A) and retraction duration (2.14 s), falling within the standard deviation for both measures in the animal data. On the other hand, biting-like patterns can also be observed in isolated ganglia of animals. However, due to the removal of sensory feedback, the *in vitro* patterns become three times longer than *in vivo* biting patterns (15.66 ± 7.34 s, Fig. 10B). The duration mainly increases in the protraction phase (10.40 ± 5.78 s) rather than in the retraction phase (4.99 ± 3.28 s). When the neural circuitry model is isolated from the proprioceptive feedback, it also produces a biting-like behavior with a substantially longer protraction phase (11.84 s, Fig. 10B) and moderately longer retraction phase (5.77 s), both of which fall within the standard deviation of the animal data.

Moreover, animals and the neuromuscular model exhibit similar changes in retraction duration when the load in swallowing increases. Under unloaded conditions, animals feed on fragile seaweed and swallow with a relatively short retraction phase (1.94 ± 0.36 s, Fig. 10C). The retraction duration obtained from the simulation (1.93 s, Fig. 10C) is close to the animal data if the model is allowed to interact with an unloaded seaweed. Under loaded conditions, animals feed on strips of seaweed that are unlikely to break during swallowing. Mechanically reinforced seaweed generates a higher load and slows down the retraction phase, increasing the retraction duration by about 52.6% (2.96 ± 0.69 s, Fig. 10D). In the simulation, fixing one end of the seaweed during swallowing increases the retraction duration, which follows the trend observed in the animal data, although the change is larger with duration increasing by about 138% (4.61 s, Fig. 10D).

**Fig. 11:**
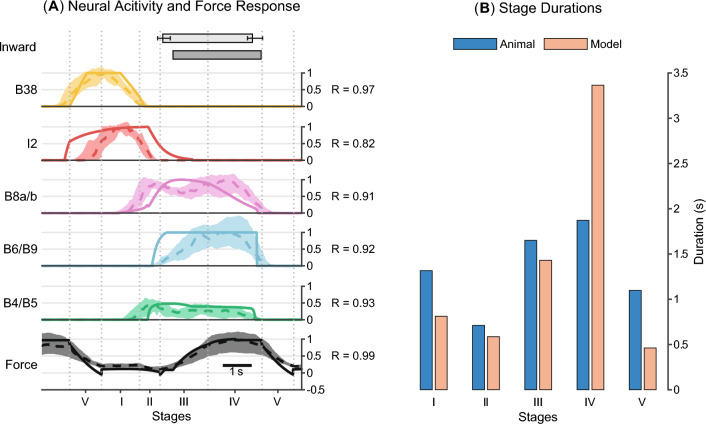
The responses of the neuromuscular model and animal subjects (Gill and Chiel 2020) in loaded swallowing experiments are shown: **A** timing of inward movement, neural activities, and normalized force on the transducer. **B** Durations of the five stages defined in Gill and Chiel (2020). Animal responses (dashed lines: mean values, shaded area: the lower and upper quartiles) are normalized with respect to the maximum of the mean values for each neuron and force, except B4 activity which is scaled by 30 Hz. The model responses (solid lines) are scaled by their maximum values, except B4 activity which is scaled by 1. Both animal and model responses are segmented by the time normalization procedure so that they can be compared with each other. Vertical dotted lines indicate the boundaries of segmentation used for normalization. For model output, the inward movement timing was invariant after normalization, so whiskers are omitted. In contrast, the animal dataset contains recording from 5 animals; inward movement was therefore variable after normalization, and whiskers are shown to reflect the variations. Cross-correlation (*R*) between the model response and animal is shown for the traces

A quantitative comparison between the simulation results and animal data further demonstrates the neuromuscular model generates animal-like kinematics, dynamics, and neural activity patterns during loaded swallowing (Fig. 11). According to the timing of certain events identified from the force record, a swallowing cycle can be segmented into five stages. A time normalization procedure (Gill and Chiel 2020) is applied to the retraction start and stop times, force time series, muscle activity series, as well as neural activity series so that records from different animals and the model could be aligned at the boundaries between the stages. It allows a direct comparison between model response and animal data using metrics like cross-correlation (*R*). With the exception of I2 muscle activity, the correlation coefficients between simulated and observed patterns are above 0.9 (Fig. 11A). Compared with animal data, the I2 muscle activation produced by the model starts early and ends late in the protraction phase. The model also tends to activate B6 early in the retraction phase. We also test if the model and animals produce similar cycle duration and stage durations without the time normalization procedure. The results (Fig. 11B) demonstrate that the Stage IV duration produced by the model is longer than the animal data, while all other stages produced by the model are shorter than the corresponding duration of animals.

**Fig. 12:**
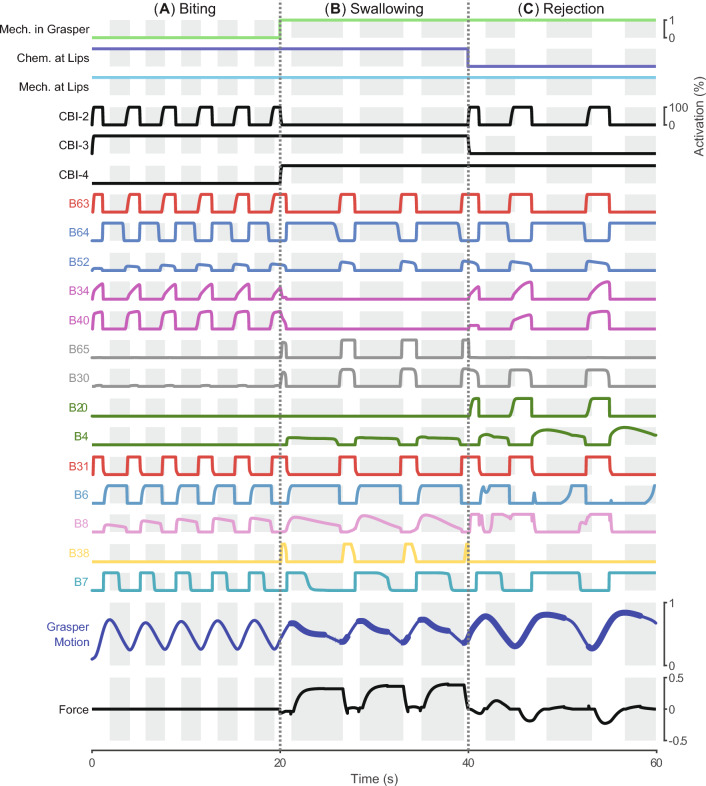
The neuromuscular model can switch from biting to swallowing and from swallowing to rejection. Initially, the interactive mechanism between the periphery and the seaweed is removed to indicate the seaweed is not yet grasped. Mechanical and chemical stimuli are present at the lips, but no mechanical stimuli are in the grasper; the network produces biting-like behavior. The behavior switches to swallowing at the first dashed vertical line, which represents a step change in the mechanical stimuli in the grasper and the restoration of the interactive mechanism. At the second vertical dashed line, the model experiences a loss of chemical stimuli at the lips and switches to rejection-like behavior

### Behavioral switching

In addition to being able to generate biting, swallowing, and rejection behaviors individually, the neuromuscular model can also adaptively switch between these behaviors based on exteroceptive feedback (Fig. 12), as observed in animals. *Aplysia* can immediately transition from biting to swallowing once they successfully grasp the food and sense a mechanical stimulus in its feeding apparatus. In the simulation, we mimic a successful grasp by applying a step change to the mechanical stimulation in the grasper and restoring the interactive mechanism between the periphery and the seaweed (the first dotted line in (Fig. 12). As a result, the model switches from biting-like behavior (Fig. 12A) driven by CBI-2 to swallowing-like behavior (Fig. 12B) driven by CBI-4. The activation of B8 is in-phase with the retraction phase so that the radula can apply inward force on the seaweed. Similarly, *Aplysia* starts producing egestive behaviors when it cannot sense food stimulus at its lips during swallowing. Such a transition can be observed in the simulation when the chemical stimulation is removed, while mechanical stimulation is maintained in the grasper and at the lips (the second dotted line in Fig. 12). As a consequence, the model transitions from swallowing to CBI-3 driven rejection behavior (Fig. 12C). The B20/B4 subnetwork mediates the phasing of the radula closure so that the seaweed can be pushed out of the feeding apparatus.

## Discussion

In this work, we have presented a model of the neural circuitry involved in controlling *Aplysia* feeding behaviors. The synaptic connections in the model are based on existing literature, and neuronal dynamics are represented using the Synthetic Nervous Systems framework. We organized the motor neurons and interneurons into three layers and five subnetworks that realize functions essential in radula protraction and retraction (Figs. 4 and 5). By optimizing the neural parameters, we found the SNS neurons can reproduce the firing threshold, current-activation relationship, and other intrinsic dynamics of particular *Aplysia* neurons (Figs. 6 and 7). We then implemented the *Aplysia* neural circuitry in a neuromuscular model with simplified peripheral mechanics (Fig. 3). The neuromuscular model is sufficient to generate three *Aplysia* feeding behaviors qualitatively (Figs. 8 and 12). The comparisons between the simulation results and reported data also suggest that the ingestive feeding behaviors generated by the neuromuscular model share similar features with animals (Figs. 9 and 11).

### Neuronal model selection

To understand the biophysical properties of neurons and how they contribute to information processing in animal brains, scientists have proposed various mathematical models of neuronal computation. Model developers may incorporate different levels of details and abstraction in models according to their specific purposes. For example, neuroscientists who want to reproduce neural dynamics as accurately as possible may adopt morphologically realistic models (Herz et al. 2006). With thousands of electrically coupled Hodgkin-Huxley-type compartments, these models have the capability to capture phenomena such as spike generation and propagation in axons (Rhodes and Llinás 2005), as well as synaptic integration in complex dendritic trees (Golding et al. 2001). However, incorporating multiple compartments and voltage-gated ion channels often leads to computational complexity issues, impeding their application in theoretical analysis or large-scale network models. Phenomenological models with a greatly reduced set of parameters can somewhat overcome this drawback. By simplifying or neglecting the majority of intrinsic mechanisms, they can achieve low computational cost but still capture fundamental neuronal behaviors (Sharpee et al. 2006). The Boolean model used in Webster-Wood et al. (2020) is a characteristic example that can represent on/off behaviors of neuron bursts, while its simulation speed can be two to three orders of magnitude faster than real-time. The drawback of this network model is that it can be too oversimplified to capture how intrinsic dynamics contribute to behaviors (see Sect. 4.2). Synthetic Nervous Systems provide a compromise in model complexity between these two extremes.

In this work, we modeled neurons using the Synthetic Nervous System framework. Each neuron is represented by a single compartment with biologically plausible elements. We neglected the spiking mechanisms, abstracting axons as activation functions. This simplification is based on the hypothesis that the generation of spikes in neurons is a trade-off between the coding efficiency and the need for signal transmission (Koch 1998). Due to this simplification, the simulation speed of SNS neurons is typically one order of magnitude faster than spiking neuron models. Our results (see Sect. 3.1) suggest that SNS neurons can reflect the intrinsic dynamics of *Aplysia* neurons (Figs. 6 and 7). Furthermore, implementing them in neural circuitry can generate an animal-like pattern generator (Fig. 10B). Although this simplification does successfully reproduce intrinsic dynamics and multifunctional feeding when coupled with a biomechanical model, it is possible that spike timing is critical for control in the animal. Therefore, more tests need to be conducted to investigate the effect of such spiking mechanisms in feeding in our model. Such mechanisms could be added to the SNS framework if the spike timing is found to be critical (Szczecinski et al. 2020). Recent related work also implies using single-compartment models to express the rich computation performed in dendritic trees is possible (Li et al. 2019, 2023) and this approach could be integrated into the SNS in future studies.

### Central pattern generators and feedback pathways

Central pattern generators (CPGs) and feedback pathways are regarded as two primary neural control mechanisms underlying the rhythmic behaviors in animals (Song and Geyer 2015). A CPG is a set of neurons that can generate cyclic outputs without rhythmic inputs (Ijspeert 2008). Experimental observations have verified the existence of CPGs in many animals (Fedirchuk et al. 1998; Grillner 1975). Stimulation to the isolated spinal cords of some vertebrates, including lampreys and salamanders, can elicit rhythmic patterns called fictive locomotion (Ijspeert 2008; Cohen and Wallén 1980). These motor patterns resemble the behavioral patterns observed in intact animals, implying CPGs play a central role in their locomotion control. It was assumed that CPGs were also essential for human locomotion (Taga 1995), but recent gait disturbance experiments reveal that CPGs alone cannot explain human reactions to a range of unexpected disturbances during normal walking (Sloot et al. 2015; Rafiee and Kiemel 2020). On the contrary, the evidence supports that feedback pathways are a critical component of overall locomotion control.

It is likely that both CPGs and feedback pathways contribute to the feeding control of *Aplysia*. Previous studies (Jing and Weiss 2001; Jing et al. 2004) have shown that stimulating specific CBI neurons in the isolated ganglia can initiate motor patterns corresponding to feeding-related behaviors. In this work, we verified that SNS neurons with animal like intrinsic dynamics are sufficient to constitute a neural circuitry that generates cyclic patterns without feedback input. When feedback loops are removed, the circuitry model can generate biting-like patterns sharing similar oscillatory timing with those observed in the isolated ganglia (Fig. 10B). It should be noted, however, that the timing of motor patterns in ganglia without feedback is far from similar to feeding behaviors generated by intact animals. The elicited biting-like patterns in the isolated ganglia can be three times longer than *in vivo* biting (Fig. 10A, B). We integrated the SNS neural circuitry with a peripheral biomechanical model and found the ingestive behaviors produced by the combined neuromuscular model are more consistent with *in vivo* observation (Figs. 10 and 11). These results indicate that sensory feedback may play important roles in shaping and modulating *Aplysia* feeding behaviors (Cullins et al. 2015).

### Network models of *Aplysia* feeding

Several network models have been presented to explain different aspects of the neural control underlying *Aplysia* feeding. To study how plasticity contributes to memory expression, Costa et al. (2020) developed a reduced model of the CPG circuit in *Aplysia*. With conductance-based neurons and their synaptic connections, the model can produce ingestion-like, rejection-like, and intermediate buccal motor patterns. The model focused on the intrinsic features of the circuit, ignoring the effects of feedback signals on the neural activities and behaviors. Lyttle et al. (2017) explored the interaction between neural circuits and biomechanics in motor control by analyzing a neuromechanical model of *Aplysia* feeding. The neural network part of the model included three neural pools corresponding to three ingestion phases. Each of these neural pools receives sensory feedback from biomechanics and inhibitory inputs from other neural pools. Although the above models can generate multiple modes of cyclic patterns, they cannot achieve active switching among these modes. To study control for multifunctionality in *Aplysia* feeding, Webster-Wood et al. (2020) incorporated key neurons, including higher-order interneurons in the cerebral ganglion, into their Boolean network model. Driven by sensory feedback, the model can flexibly switch behaviors as external stimuli vary.

We developed a SNS network to capture essential features of the *Aplysia* feeding circuit. The model includes neurons, synapses, and feedback pathways identified in existing literature to ensure biological plausibility. It can independently produce three behaviors of interest through the local coordination of buccal interneurons. The global coordination mediated by the CBI neurons further allows the transition among these behaviors. It can demonstrate the contribution of both intrinsic dynamics and sensory feedback. Similar to the CPG model presented in Costa et al. (2020), it is sufficient to produce oscillatory modes without sensory feedback. The Boolean network in Webster-Wood et al. (2020) cannot reflect such motor patterns. Due to simplifying the neuronal dynamics, it will stop working once sensory inputs are removed. When combined with a biomechanical model, the incorporated feedback pathways in the SNS network provide excitatory or inhibitory inputs to specific motor neurons and interneurons, thereby allowing the generation of animal-like feeding behaviors.

### Limitations and future work

Although we showed the integration of the presented neural circuitry model and a previous biomechanical model can qualitatively produce animal-like feeding behaviors, there are some discrepancies between the simulation results and animal data. In loaded swallowing, the retraction duration of the neuromuscular model does not lie within the range found in animal experiments (Fig. 10). The largest discrepancy between the simulated and observed activities occurs in the I2 muscle ($R = 0.82$, Fig. 11) whose onset is early by 14% and termination is late by 11% of the swallowing cycle. The correlation coefficient of B6 is above 0.9 (0.92), but the model response starts early in stage II. In contrast, the B6 activity observed in animals (mean value) does not achieve 90% of its maximum value until the end of stage III. The model also tends to produce different stage durations. In particular, stage IV of the model is 79% longer than observed in animals, while stages I, II, and III of the model are 41%, 17%, and 52% shorter than observed in animals, respectively. The model might need to incorporate more biological details to increase the quality of the match for kinematics, dynamics, and neural activities.

In this work, the neuronal parameters of B64, B4, B34, and B52 were estimated by optimization algorithms, while other parameters, such as synaptic parameters (Table 3), were found by hand-tuning (see Sect. 2.4). Since the neural circuitry model contains hundreds of parameters, the hand-tuning process was tedious and time-consuming. In addition, the hand-tuned parameters are not necessarily the desired values. The quality of the model can be further improved with more effective parameter exploration methods. In future work, we will automate the parameter search by using advanced optimization or machine learning algorithms, such as Markov Chain Monte Carlo (MCMC) methods (van Ravenzwaaij et al. 2018). MCMC is a Bayesian inference method that has been leveraged to estimate key parameters in complex neuron models (Wang et al. 2022). It can not only provide a point estimate of a specific parameter set, but also efficiently reveal the entire landscape of the fitness functions. Therefore, it has an advantage in quantifying the parameter sensitivity that is not analyzed in the work presented here.

In actual feeding behaviors, the movement of the grasper or head is more complex than rigid translation along a single dimension. Experimental results, including *in vivo* magnetic resonance images (Novakovic et al. 2006), suggest that the closed grasper rotates about the hinge muscle during Type B rejection. The grasper even changes its shape during opening and closing, leading to a mechanical reconfiguration of the muscular system (Ye et al. 2006). Kinematics models (Neustadter et al. 2002), kinetic models (Novakovic et al. 2006), and experimental results (Ye et al. 2006) have demonstrated that these grasper movements can be critical for the neural control of multifunctionality. The work reported in this paper integrated the neural circuitry model with a simplified biomechanical model. As a consequence, while the model can qualitatively generate multifunctional feeding behaviors, it fails to capture the interaction between neural control and grasper rotation or deformation. Future iterations of the model will be integrated with more detailed biomechanical models to show how the combination of neural control and peripheral mechanics contributes to the generation of feeding behaviors.

Intermediate behaviors and cycle-to-cycle variability are not explored in this work. In fact, biting, swallowing, and rejection that can be qualitatively reproduced by the model only constitute a small group of *Aplysia* feeding behaviors. According to external sensory cues, animals can selectively generate ingestive, egestive, and various intermediate behaviors along a behavioral continuum (Katzoff et al. 2006). Previous literature (Morton and Chiel 1993a) has demonstrated that those intermediate behaviors, such as the attempt to reposition the food and retry swallowing before rejection, are vital for effective feeding. Since SNS neural circuitry can generate graded rather than on/off activity, it has the potential to capture these behaviors as well. Further development of the simulation needs to be done to investigate intermediate feeding behaviors controlled by the present model. On the other hand, animals tend to generate behaviors with variability (Cullins et al. 2015). Even within a given individual, the observed behaviors have different periods and magnitudes in each cycle. The variability in motor control may contribute to the success of behaviors and animal survival (Marder and Taylor 2011). Stochastic parameters can be incorporated into the model to capture the important variability (Costa et al. 2020).

## Figures and Tables

**Fig. 13:**
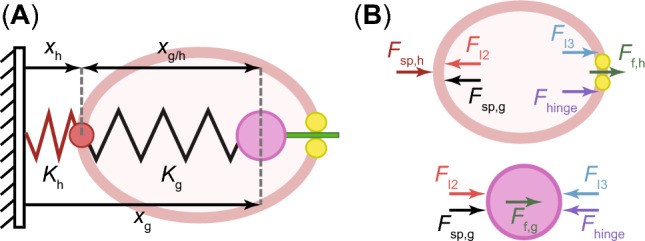
Peripheral mechanics. **A** A mass-spring-damper representation of the biomechanical model. The body-head connection and the head-grasper connection are viewed as prismatic joints with internal stiffness. Viscous damping is also incorporated to model the damping between the segments and seawater. **B** Free body diagrams showing all possible muscle forces, friction forces, and spring forces on the head segment (top) and grasper segment (bottom)

## Data Availability

Data and code are available at https://doi.org/10.5281/zenodo.10228820.

## Biomechancial model

### Peripheral mechanics

The peripheral mechanics of the biomechanical model represents a simplified *Aplysia* feeding system with two segments connected by two translational joints and actuated by four muscles. Prismatic joints connect the segments with one DOF between the head and body and another DOF between the grasper and head (Fig. 13A). Three muscle units, including the I2 protractor muscle, the I3 retractor muscle, and the hinge retractor muscle actuate the head-grasper joint. The remaining muscle units, the I4 muscle and the anterior portion of the I3 jaw muscle, are responsible for radula closure and jaw closure, respectively. According to the free-body diagrams (Fig. 13b), the equations of motion of the system are as follows:

A1 $$\begin{aligned}&m_{\text {h}} \ddot{x}_{\text {h}} + c_{\text {h}} \dot{x}_{\text {h}} \nonumber \\&=F_{\text {sp,h}}-F_{\text {sp,g}}-F_{\text {I}2} + F_{\text {I}3} + F_{\text {hinge}} + F_{\text {f,h}} \nonumber \\&m_{\text {g}}\ddot{x}_{\text {h}} + c_{\text {g}}\dot{x}_{\text {g}} \nonumber \\&= F_{\text {sp,g}} + F_{\text {I}2} - F_{\text {I}3} - F_{\text {hinge}} + F_{\text {f,g}} \end{aligned}$$

where $F_{\text {sp,h}} = k_{\text {h}}\left( x_{\text {h}}^0 - x_{\text {h}}\right)$ and $F_{\text {sp,g}} = k_{\text {g}}\left( x_{\text {g/h} }^0 - x_{\text {g/h}}\right)$ are the spring force between the head and neck and between the grasper and the head of the animal, respectively. *x* denotes position, *m* denotes mass, *c* denotes damping ratio, and *k* denotes spring constant. The subscript h and g denote the head and the grasper, respectively. $x_{\text {h}}^0$ and $x_{\text {g/h}}^0$ are the resting length of the prismatic joints. $F_{\text {I}2}$, $F_{\text {I}3}$, and $F_{\text {hinge}}$ are muscle forces, while $F_{\text {fh}}$ and $F_{\text {fg}}$ are friction forces between the seaweed and the corresponding segments (see Appendix A.3).

**Table 1:** Intrinsic dynamics of neurons without extra ion channels

Neuron	Intrinsic currents	Activation function
B4		
	$I_\text {cap} = 0.01\frac{dU}{dt}$	$\min \left( 1,\max \left( 0,\frac{U-6.58}{U-25}\right) \right)$
	$$I_\text {L} = 0.2(-60-U)$$	
B6		
	$I_\text {cap} = 0.01\frac{dU}{dt}$	$\min \left( 1,\max \left( 0,\frac{U-5}{U-43.3}\right) \right)$
	$$I_\text {L} = 0.1(-60-U)$$	
B7		
	$I_\text {cap} = 0.01\frac{dU}{dt}$	$\frac{1}{1+e^{-(U+43.5)/2.5}}$
	$$I_\text {L} = 0.1(-60-U)$$	
B8		
	$I_\text {cap} = 0.01\frac{dU}{dt}$	$\min \left( 1,\max \left( 0,\frac{U-5}{U-43.3}\right) \right)$
	$$I_\text {L} = 0.1(-60-U)$$	
B30		
	$I_\text {cap} = 0.01\frac{dU}{dt}$	$\min \left( 1,\max \left( 0,\frac{U-5}{U-43.3}\right) \right)$
	$$I_\text {L} = 0.1(-60-U)$$	
B31		
	$I_\text {cap} = 0.01\frac{dU}{dt}$	$\min \left( 1,\max \left( 0,\frac{U-5}{U-43.3}\right) \right)$
	$$I_\text {L} = 0.05(-60-U)$$	
B38		
	$I_\text {cap} = 0.01\frac{dU}{dt}$	$\min \left( 1,\max \left( 0,\frac{U-5}{U-43.3}\right) \right)$
	$$I_\text {L} = 0.1(-60-U)$$	
B40		
	$I_\text {cap} = 0.01\frac{dU}{dt}$	$\min \left( 1,\max \left( 0,\frac{U-5}{U-43.3}\right) \right)$
	$$I_\text {L} = 0.1(-60-U)$$	
B63		
	$I_\text {cap} = 0.01\frac{dU}{dt}$	$\min \left( 1,\max \left( 0,\frac{U-5}{U-43.3}\right) \right)$
	$$I_\text {L} = 0.04(-60-U)$$	
CBI2		
	$I_\text {cap} = 0.01\frac{dU}{dt}$	$\min \left( 1,\max \left( 0,\frac{U-5}{U-43.3}\right) \right)$
	$$I_\text {L} = 0.1(-60-U)$$	
CBI3		
	$I_\text {cap} = 0.01\frac{dU}{dt}$	$\min \left( 1,\max \left( 0,\frac{U-5}{U-43.3}\right) \right)$
	$$I_\text {L} = 0.1(-60-U)$$	
CBI4		
	$I_\text {cap} = 0.01\frac{dU}{dt}$	$\min \left( 1,\max \left( 0,\frac{U-5}{U-43.3}\right) \right)$
	$$I_\text {L} = 0.1(-60-U)$$	

### Muscle model

The muscle force $F_{*}$ is calculated as followed

A2 $$\begin{aligned} F_{*}&= F_{*,\max }T_{*}\text {MA}_{*} \nonumber \\ \tau _{*}^2 \ddot{T}_{*} + 2\tau _{*}\dot{T}_{*}&= y_{*} \end{aligned}$$

where the subscript $*$ is the index of the muscle unit, $F_{*,\max }$ is the maximum muscle force, $\text {MA}_{*}$ is the mechanical advantage ($\text {MA}_{\text {I}2} = 1 - x_{\text {g/h}}$, $\text {MA}_{\text {I}3} = x_{\text {g/h} }$, $\text {MA}_{\text {hinge}} = \max \left( x_{\text {g/h}} - 0.5,0\right)$), and $y_{*}$ is the output of the corresponding motor neuron. The activation of the corresponding muscle, $T_{*}$, is determined by a cascade of two first order systems with time constant $\tau _{*}$.

### Grasper-seaweed interaction and seaweed movement

The biomechanical system can interact with the environment via two points. The first contact point is between the grasper and its food, seaweed. With a stimulus sent to the I4 muscle, the grasper closes and applies contact force to the food. The other contact point is located at the jaw. Contraction of the anterior portion of the I3 muscle makes the jaw pinch the seaweed. $P_{\text {g}}$, the normal contact between the grasper and food, and $P_{\text {h}}$, the normal contact force between the head and food, are calculated similarly to Eq. (A2)

A3 $$\begin{aligned} \tau _{*}^2 \ddot{P}_{*} + 2\tau _{*}\dot{P}_{*} = y_{*} \end{aligned}$$

where the subscript $*$ denoting h or g is the index of the contact point, $\tau _*$ is the time constant of the corresponding muscle, $y_{*}$ is the output of motor neurons innvervating the corresponding muscle ($y_g = y_{\text {I4}}$, $y_{\text {I3,ant}} = y_{\text {B6}} + y_{\text {B38}}$).

The tangential contact force acts like the Coulomb friction model,

A4 $$\begin{aligned} |F_{f,*}| \le \mu _{\text {s},*} P_{*},&\qquad \text {static case} \nonumber \\ |F_{f,*}| = \mu _{\text {k},*} P_{*},&\qquad \text {sliding case} \end{aligned}$$

**Table 2:** Intrinsic dynamics of neurons with extra ion channels

Neuron	Intrinsic currents	Dynamic variables	Steady states	Relaxtion time (ms)	Activation function
B20					
	$I_\text {cap} = 0.01\frac{dU}{dt}$				$\frac{1}{1+e^{-(U+43.5)/2.5}}$
	$$I_\text {L} = 0.1(-60-U)$$				
	$I_\text {K} = -1.25A^2B(60+U)$	$\frac{dA}{dt} = \frac{A_\infty - A}{\tau _A}$	$A_\infty = \frac{1}{1+e^{-60-U}}$	$\tau _A = 0.02+\frac{0.18}{\left[ 1+e^{56+U}\right] ^2}$	
		$\frac{dB}{dt} = \frac{B_\infty - B}{\tau _B}$	$B_\infty = \frac{1}{\left[ 1+e^{55+U}\right] ^2}$	$\tau _B = 0.5$	
B34					
	$I_\text {cap} = 0.01\frac{dU}{dt}$				$\min \left( 1,\max \left( 0,\frac{U-5}{U-43.3}\right) \right)$
	$$I_\text {L} = 0.1(-60-U)$$				
	$I_\text {K} = -1.25A^2B(60+U)$	$\frac{dA}{dt} = \frac{A_\infty - A}{\tau _A}$	$A_\infty = \frac{1}{1+e^{-60-U}}$	$\tau _A = 0.02+\frac{0.18}{\left[ 1+e^{56+U}\right] ^2}$	
		$\frac{dB}{dt} = \frac{B_\infty - B}{\tau _B}$	$B_\infty = \frac{1}{\left[ 1+e^{55+U}\right] ^2}$	$\tau _B = 0.5$	
B52					
	$I_\text {cap} = 0.01\frac{dU}{dt}$				$\min \left( 1,\max \left( 0,\frac{U-1.96}{U-51.7}\right) \right)$
	$$I_\text {L} = 0.1(-62-U)$$				
	$I_\text {Na} = 0.1A(30-U)$	$\frac{dA}{dt} = \frac{A_\infty - A}{\tau _A}$	$A_\infty = \frac{1}{\left[ 1+e^{61+U}\right] ^3}$	$\tau _A = 5+\frac{15}{1+e^{\frac{60+U}{5}}}$	
B64					
	$I_\text {cap} = 0.01\frac{dU}{dt}$				$\frac{1}{1+e^{-(U+43.5)/2.5}}$
	$$I_\text {L} = 0.19(-60-U)$$				
	$I_\text {Na} = 0.1A(30-U)$	$\frac{dA}{dt} = \frac{A_\infty - A}{\tau _A}$	$A_\infty = \frac{1}{1+e^{-50-U}}$	$\tau _A = 0.1$	
	$I_\text {K} = -0.35A(70+U)$	$\frac{dA}{dt} = \frac{A_\infty - A}{\tau _A}$	$A_\infty = \frac{1}{1+e^{-50-U}}$	$\tau _A = 4$	
B65					
	$I_\text {cap} = 0.01\frac{dU}{dt}$				$\frac{1}{1+e^{-(U+43.5)/2.5}}$
	$$I_\text {L} = 0.08(-60-U)$$				
	$I_\text {Na} = -0.3A(40+U)$	$\frac{dA}{dt} = \frac{A_\infty - A}{\tau _A}$	$A_\infty = \frac{1}{\left[ 1+e^{\frac{-45-U}{5}}\right] ^3}$	$\tau _A = 2$	
	$I_\text {K} = -0.01A(60+U)$	$\frac{dA}{dt} = \frac{A_\infty - A}{\tau _A}$	$A_\infty = \frac{1}{\left[ 1+e^{\frac{40+U}{5}}\right] ^4}$	$\tau _A = 1$

**Table 3:** Summary and parameters of synapses

Postsynaptic	Presynaptic	Types	(Maximal)	Reversal	Time
			Conductance	Potential	Constants
			($$\mu $$S)	(mV)	(ms)
B4					
	B20	Slow excitatory	$-$0.3	– 60	2(*r*), 5(*s*)
	B31	Electrical	0.008		
	B64	Fast excitatory	0.1	0	0.01(*r*), 0.01(*s*)
		Electrical	0.008		
B6					
	B4	Fast inhibitory	2	– 80	0.01(*r*), 0.01(*s*)
	B63	Fast inhibitory	5	– 80	0.01(*r*), 0.01(*s*)
	B64	Fast excitatory	0.4	0	0.25(*r*), 0.25(*s*)
B7					
	B64	Fast excitatory	0.4	0	0.01(*r*), 0.01(*s*)
B8					
	B4	Fast inhibitory	5	– 80	0.015(*r*), 0.015(*s*)
	B20	Fast excitatory	3.5	0	0.004(*r*), 0.0015(*s*)
		Slow excitatory	0.9	0	1(*r*), 1(*s*)
	B30	Slow excitatory	$-$0.15	– 60	3(*r*), 3(*s*)
		Fast inhibitory	0.5	– 70	0.2(*r*), 0.001(*s*)
	B34	Slow excitatory	$-$0.03	– 65	1(*r*), 1(*s*)
		Fast inhibitory	0.2	– 80	0.01(*r*), 0.01(*s*)
	B40	Slow excitatory	0.3	0	5(*r*), 0.001(*s*)
		Fast inhibitory	0.4	– 75	0.015(*r*), 0.015(*s*)
	B64	Fast excitatory	0.002	0	0.01(*r*), 0.01(*s*)
	B65	Fast excitatory	0.06	0	0.004(*r*), 0.0015(*s*)
		Slow excitatory	0.3	0	1(*r*), 1(*s*)
B20					
	CBI2	Slow excitatory	0.06	0	0.5(*r*), 0.5(*s*)
	CBI3	Fast inhibitory	2	– 80	0.01(*r*), 0.01(*s*)
	CBI4	Slow excitatory	0.06	0	0.5(*r*), 0.5(*s*)
	B31	Electrical	0.004		
	B34	Fast excitatory	0.16	0	0.2(*r*), 0.2(*s*)
	B63	Fast excitatory	0.2	0	0.01(*r*), 0.01(*s*)
		Slow excitatory	0.1	0	0.5(*r*), 0.5(*s*)
		Electrical	0.004		
	B64	Fast inhibitory	2	– 80	0.01(*r*), 0.01(*s*)
	B65	Fast excitatory	0.12	0	0.05(*r*), 0.05(*s*)
		Slow excitatory	$-$0.01	– 60	0.5(*r*), 0.5(*s*)
		Electrical	0.004		
B30					
	CBI4	Fast excitatory	0.1	0	0.01(*r*), 0.01(*s*)
		Slow excitatory	0.1	0	0.5(*r*), 5(*s*)
	B31	Electrical	0.009		
	B63	Electrical	0.006		
	B64	Fast inhibitory	5	– 80	0.01(*r*), 0.01(*s*)
	B65	Fast excitatory	0.05	0	0.01(*r*), 0.01(*s*)
B31					
	CBI2	Fast excitatory	0.02	0	0.001(*r*), 0.001(*s*)
		Slow excitatory	0.002	0	0.5(*r*), 5(*s*)
	B4	Electrical	0.008		
	B20	Electrical	0.004		
	B30	Fast excitatory	0.05	0	0.005(*r*), 0.005(*s*)
		Electrical	0.009		
	B34	Fast excitatory	0.125	0	0.01(*r*), 0.01(*s*)
		Slow excitatory	0.05	0	1.5(*r*), 1.5(*s*)
	B63	Fast excitatory	1	0	0.01(*r*), 0.01(*s*)
		Slow excitatory	0.4	0	0.2(*r*), 0.2(*s*)
		Electrical	0.015		
	B64	Fast inhibitory	0.5	-80	0.01(*r*), 0.01(*s*)
	B65	Fast excitatory	0.1	0	0.004(*r*), 0.0015(*s*)
		Electrical	0.004		
B34					
	CBI2	Fast excitatory	0.3	0	0.01(*r*), 0.01(*s*)
	B40	Electrical	0.008		
	B63	Fast excitatory	0.03	0	0.015(*r*), 0.015(*s*)
	B64	Fast inhibitory	2	– 80	0.1(*r*), 0.1(*s*)
	B65	Electrical	0.008		
B38					
	B20	Slow inhibitory	2	– 80	0.5(*r*), 0.5(*s*)
	B40	Fast inhibitory	5	– 80	0.01(*r*), 0.01(*s*)
	B64	Fast inhibitory	5	– 80	0.1(*r*), 0.1(*s*)
B40					
	CBI2	Fast excitatory	0.2	0	0.01(*r*), 0.01(*s*)
		Slow excitatory	0.2	0	0.2(*r*), 2(*s*)
	B34	Electrical	0.008		
	B63	Electrical	0.004		
	B64	Fast inhibitory	2	– 80	0.01(*r*), 0.01(*s*)
	B65	Slow inhibitory	4	– 60	1.5(*r*), 1.5(*s*)
		Electrical	0.008		
B52					
	B64	Fast inhibitory	3	– 80	0.01(*r*), 0.01(*s*)
B63					
	CBI2	Fast excitatory	0.06	0	0.01(*r*), 0.01(*s*)
		Slow excitatory	0.06	0	0.5(*r*), 5(*s*)
	CBI4	Fast excitatory	0.06	0	0.01(*r*), 0.01(*s*)
		Slow excitatory	0.06	0	0.5(*r*), 5(*s*)
	B20	Electrical	0.004		
	B30	Fast excitatory	0.03	0	0.01(*r*), 0.01(*s*)
		Electrical	0.006		
	B31	Electrical	0.015		
	B34	Fast excitatory	0.3	0	0.025(*r*), 0.025(*s*)
	B40	Electrical	0.004		
	B64	Fast inhibitory	2	– 80	0.01(*r*), 0.01(*s*)
	B65	Fast excitatory	0.1	0	0.004(*r*), 0.0015(*s*)
		Electrical	0.006		
B64					
	B4	Electrical	0.008		
	B30	Slow excitatory	0.05	0	0.5(*r*), 0.5(*s*)
		Fast inhibitory	0.02	– 80	0.02(*r*), 0.02(*s*)
	B31	Fast inhibitory	0.1	– 80	0.1(*r*), 0.1(*s*)
	B34	Fast inhibitory	0.15	– 80	0.2(*r*), 0.2(*s*)
	B40	Fast inhibitory	0.18	– 65	0.015(*r*), 0.015(*s*)
	B52	Fast inhibitory	5.6667	– 80	0.05(*r*), 0.05(*s*)
	B63	Slow excitatory	0.2	0	0.2(*r*), 0.2(*s*)
		Fast inhibitory	0.02	– 80	0.15(*r*), 0.15(*s*)
	B65	Fast excitatory	0.05	0	0.004(*r*), 0.0015(*s*)
		Slow inhibitory	0.03	– 80	0.5(*r*), 0.5(*s*)
B65					
	CBI2	Fast inhibitory	0.9	– 80	0.01(*r*), 0.01(*s*)
		Slow inhibitory	0.9	– 80	0.5(*r*), 5(*s*)
	CBI4	Slow inhibitory	0	– 80	0.5(*r*), 5(*s*)
	B20	Electrical	0.004		
	B31	Electrical	0.004		
	B34	Electrical	0.008		
	B40	Electrical	0.008		
	B63	Slow excitatory	0.4	0	0.1(*r*), 0.1(*s*)
		Electrical	0.006		
	B64	Fast inhibitory	2	– 80	0.01(*r*), 0.01(*s*)

where the direction of the friction force is opposite to the relative motion, $*$ denotes the contact point as above, $\mu _{\text {s},*}$ and $\mu _{\text {k},*}$ are the corresponding static and dynamic coefficient of friction, respectively (see Webster-Wood et al. (2020) for how to determine the type and direction of friction force).

## Neuromuscular parameters

See the Tables 1, 2, 3, 4 and 5.

**Table 4:** Synaptic thresholds of synapses from B4

Postsynaptic neuron	Synaptic threshold
B6	0.55
B8	0.5

**Table 5:** Summary of feedback pathways

Neuron	Type	Feedback currents
B64		
	Proprioceptive	$I_{\text {app},1} = \max \left( 200 (x_{\text {g/h}} -0.38),0\right)$
	Proprioceptive	$I_{\text {app},2} = -\max \left( -200 (x_{\text {g/h}} -0.38),0\right)$
B4		
	Proprioceptive	$I_{\text {app},1} = \max \left( 20 (x_{\text {g/h}} -0.7),0\right)$
	Exteroceptive	$I_{\text {app},2} = \max \left( -20 (\text {ME}_\text {g} -1),0\right)$
B7		
	Proprioceptive	$I_{\text {app},1} = \max \left( 50 (x_{\text {g/h}} -0.5),0\right)$
	Proprioceptive	$I_{\text {app},2} = -\max \left( -100 (x_{\text {g/h}} -0.7),0\right)$
B38		
	Proprioceptive	$I_{\text {app},1} = \max \left( -25 (x_{\text {g/h}} -0.7),0\right)$
	Exteroceptive	$I_{\text {app},2} = \max \left( -20 (\text {CH}_\text {l} -1),0\right)$
	Exteroceptive	$I_{\text {app},3} = \max \left( -20 (\text {ME}_\text {l} -1),0\right)$
	Exteroceptive	$I_{\text {app},4} = \max \left( -20 (\text {ME}_\text {g} -1),0\right)$
